# Changes of Sand Fly Populations and *Leishmania infantum* Infection Rates in an Irrigated Village Located in Arid Central Tunisia

**DOI:** 10.3390/ijerph13030329

**Published:** 2016-03-16

**Authors:** Walid Barhoumi, Wasfi Fares, Saifedine Cherni, Mohamed Derbali, Khalil Dachraoui, Ifhem Chelbi, Marcelo Ramalho-Ortigao, John C. Beier, Elyes Zhioua

**Affiliations:** 1 Laboratory of Vector Ecology, Pasteur Institute of Tunis, 13 Place Pasteur BP 74, Tunis 1002, Tunisia; walidbarhoumi.ipt2009@yahoo.fr (W.B.); fwasfi@yahoo.fr (W.F.); saifcherni@yahoo.com (S.C.); derbali.hiba@gmail.com (M.D.); khalil.dachraoui@yahoo.com (K.D.); ifhemc2001@yahoo.fr (I.C.); 2 Laboratory of Transmission, Control and Immunobiology of Infections, Pasteur Institute of Tunis, Tunis 1002, Tunisia; 3 Faculty of Sciences of Bizerte, University of Carthage, Bizerte 7021, Tunisia; 4 Department of Entomology, Kansas State University, Manhattan, KS 66506, USA; mortigao@ksu.edu; 5 Department of Public Health Sciences, University of Miami Miller School of Medicine, Miami, FL 33136, USA; jbeier@med.miami.edu

**Keywords:** zoonotic visceral leishmaniasis, irrigation, sand flies, *Phlebotomus perniciosus*, *Phlebotomus perfiliewi*, *Phlebotomus longicuspis*, *Leishmania infantum*, emergence of ZVL, North Africa

## Abstract

The current spread of zoonotic visceral leishmaniasis (ZVL) throughout arid areas of Central Tunisia is a major public health concern. The main objective of this study is to investigate whether the development of irrigation in arid bio-geographical areas in Central Tunisia have led to the establishment of a stable cycle involving sand flies of the subgenus *Larroussius* and *Leishmania infantum*, and subsequently to the emergence of ZVL. Sand flies were collected from the village of Saddaguia, a highly irrigated zone located within an arid bio-geographical area of Central Tunisia by using modified Centers for Diseases Control (CDC) light traps. Morphological keys were used to identify sand flies. Collected sand flies were pooled with up to 30 specimens per pool according to date and tested by nested Polymerase Chain Reaction (PCR) DNA sequencing from positive pools was used to identify *Leishmania* spp. A total of 4915 sand flies (2422 females and 2493 males) were collected from Saddaguia in September and in October 2014. Morphological identification confirmed sand flies of the subgenus *Larroussius* to be predominant. PCR analysis followed by DNA sequencing indicated that 15 pools were infected with *L. infantum* yielding an overall infection rate of 0.6%. The majority of the infected pools were of sand fly species belonging to subgenus *Larroussius*. Intense irrigation applied to the arid bio-geographical areas in Central Tunisia is at the origin of the development of an environment capable of sustaining important populations of sand flies of the subgenus *Larroussius*. This has led to the establishment of stable transmission cycles of *L. infantum* and subsequently to the emergence of ZVL.

## 1. Introduction

Zoonotic visceral leishmaniasis (ZVL) caused by *Leishmania infantum*, and transmitted by sand fly species of the subgenus *Larroussius* is endemic in the Western Mediterranean basin [1]. Most vectors incriminated in the transmission of *L. infantum* in the Old World belong to the subgenus *Larroussius* [2]. In North Africa, *Phlebotomus perniciosus*, *Phlebotomus perfiliewi* and *Phlebotomus longicuspis* are the principal vectors of *L. infantum* [3,4,5,6]. Each year in Tunisia, between 100 and 160 cases of ZVL are reported, the majority in children younger than 5 years old [6,7]. Until the 1980s, cases of ZVL in Tunisia were limited to the northern humid, sub-humid and semi-arid areas [8,9,10,11]. However, more recently, ZVL has become endemic in arid areas located in Central Tunisia following the report of several autochthonous cases [12,13,14,15,16,17]. While most of the aforementioned studies were based on epidemiological data, few entomological surveys have been performed to corroborate the epidemiological findings related to the expansion of ZVL from Northern to Central Tunisia.

We previously demonstrated that the extension of ZVL to Central Tunisia was associated with the geographical expansion of *P. perniciosus* and *P. perfiliewi* [18]. Based on geographical and remote sensing approaches, the density of *P. perfiliewi* and *P. perniciosus* was predicted to be moderately high in the arid bio-geographical areas in Central Tunisia [19]. The results of this study highlighted that abundance of *P. perfiliewi* and *P. perniciosus* is associated with the development of irrigated areas [19]. From an epidemiological point of view, the direct impact of irrigation in arid areas located in Central Tunisia on the emergence of ZVL needs to be assessed. Thus, we hypothesize that for the emergence of ZVL in the arid bio-geographical areas located in Central Tunisia, a stable cycle involving sand fly species of the subgenus *Larroussius* and *L. infantum* needs to be established following environmental changes mainly due to irrigation. In the present study, we report on sand fly species diversity and their relative abundance and infection rates with *L. infantum* within a highly irrigated area located in the arid bio-geographical region of Central Tunisia. Our data reinforce the idea that all prerequisites necessary for the emergence of new focus of ZVL are currently present in Saddaguia.

## 2. Materials and Methods

### 2.1. Study Site

**Figure 1:**
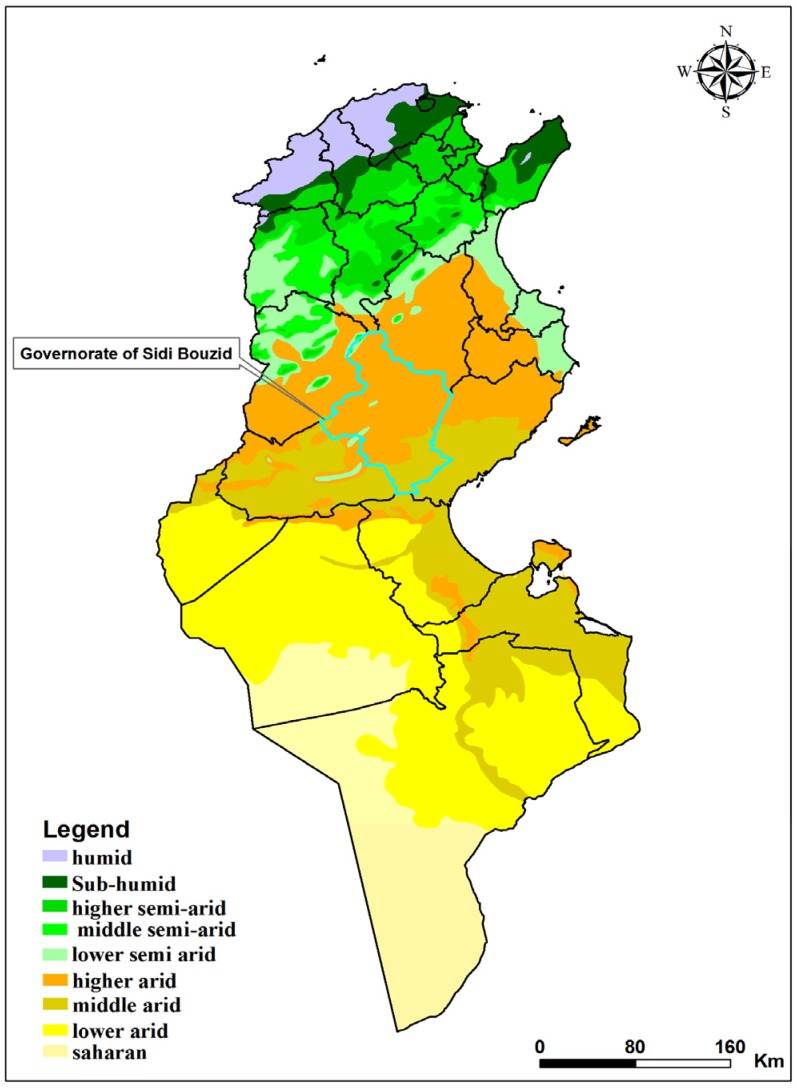
Bioclimatic map of Tunisia showing sand fly sampling sites in the governorate of Sidi Bouzid, Tunisia.

Tunisia spans a wide range of climates, from a rainy (in winter) Mediterranean climate in the North to the Saharan climate in the south. The Tunisian Ridge, running from northeast to southwest for some 220 km marks the climatic boundary between the Mediterranean north and the dry steppe of Central Tunisia, separating northern and southern Tunisia. Between the northern slopes of the Tunisian Ridge and the chains of hills bounding it on the south are extensive plateaus, called the High Tell. The Sahara is separated from the central steppe land by a series of salted areas called chotts (Figure 1).

**Figure 2:**
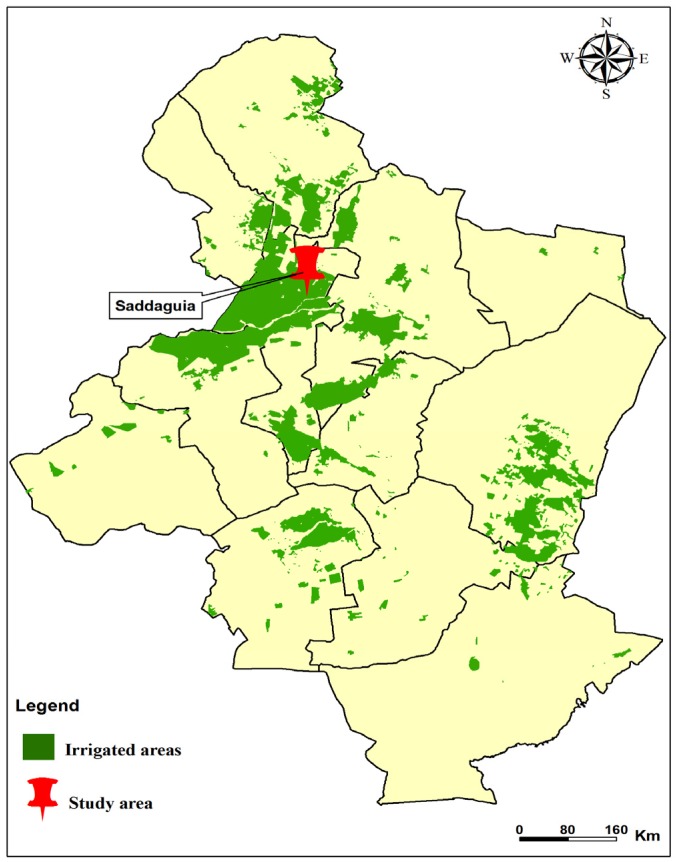
Map of the governorate of Sidi Bouzid showing irrigated areas.

Sampling of sand flies was performed in the village of Saddaguia (35°05′ N, 9°25′ E). The village of Saddaguia belongs to the governorate of Sidi Bouzid covering a surface area of 44 km^2^ and with a total population of 2815 inhabitants. The number of wells in the governorate of Sidi Bouzid increased from 2000 in 1976 to 7406 in 1987 [12]. The density of wells per km^2^ in the governorate of Sidi Bouzid increased from 0.28 wells/km^2^ in 1976 to 0.98 wells/km^2^ in 1987. It is of major importance to point out that the density of wells in Saddaguia increased from 3.53 wells/km^2^ in 1976 to 13.23 wells/km^2^ in 1987 [12]. In 1987, the density of wells in Saddaguia was 13 times higher than the rest of the governorate. Thus, the village of Saddaguia is a highly irrigated area located in the arid bio-geographical region of Central Tunisia with crop production as the main agricultural activity (Figure 2). Livestock farming mainly sheep is an important agricultural activity in the governorate of Sidi Bouzid including Saddaguia. The governorate of Sidi Bouzid is a highly endemic area with multiple foci for zoonotic cutaneous leishmanisis (ZCL) caused by *Leishmania major* and transmitted by *Phlebotomus papatasi* [3,20,21,22,23,24]. The first case of ZVL in Saddaguia was reported in 1986 followed by five other cases between 1986 and 1988 [12]. This outbreak was related to the introduction of dogs from ZVL endemic areas located in Northern Tunis by nomadic shepherds [12]. The seroprevalence observed among dogs in Saddaguia in 1987 and 1988, was 8% and 12.2%, respectively [12]. Thus, Saddaguia is considered as a limited ZVL focus area within the much larger ZCL endemic focus located in the governorate of Sidi Bouzid [12].

### 2.2. Collection and Identification of Sand Flies

Sand flies were collected from residences and animal shelters located in the peri-domicile areas of three family-owned farms using Centers for Diseases Control (CDC) light traps. Traps were placed inside houses and animal shelter including chicken coops, rabbit holes, cattle pens, and sheep pens, from dusk to dawn at each of the three sites used during one of two main sand fly activity peaks, between September and October [21,25]. Trapping of sand flies was performed during two nights in September and two nights in October. Taking into an account the number of sites (*n* = 3) and the number of biotopes per site (*n* = 5), the total number of trap nights was 180. Collected sand flies were identified individually to species level according to morphological characters [26,27]. Special attention was paid to the atypical form of *P. perniciosus* females, often misidentified as *P. longicuspis* [28,29]. Following identification, unfed female sand flies were pooled with up to a maximum of 30 specimens per pool based on date of collection, species, and biotope and then stored in 70% alcohol for molecular analysis.

### 2.3. Detection of Leishmania Spp in Female Sand Flies

Pools of female sand flies were homogenized by hand in 100 µL of phosphate buffered saline (PBS) solution using a disposable pestle. Another 100 µL of PBS were added to give a final solution of 200 µL. The mixture was clarified by centrifugation at 6000× *g* for 2 min to be used for DNA extraction with Qiagene DNA Mini Kit (Qiagen, Hilden, Germany. *L. infantum* DNA extracted previously from parasite culture was used as positive control. DNA extracted from sand fly pools was screened for the presence of *Leishmania* DNA (as a proxy for *Leishmania* infection) using a nested PCR based on a portion of the ITS-rDNA gene as described previously [30,31]. For each DNA sample extracted from pools of sand flies, an initial amplification step was performed using the Taq DNA recombinant polymerase kit (Invitrogen, Carlsbad, CA, USA in 50 µL reaction containing 5 µL 10X buffer, 3 µL MgCl_2_ (50 mM), 2 µL dNTP mix (10 mM), 1 µL of each reverse and forward primers IR1/IR2 (10 µM), 0.5 µL Taq and 10 µL of total extracted DNA. The nested PCR was carried out in 50 µL reaction containing 2 µL of the initial PCR and 48 µL of a mixture containing 5 µL 10X buffer, 3 µL MgCl_2_ (50 mM), 2 µL dNTP mix (10 mM), 1 µL of each reverse and forward internal primers ITS1F/ITS2R4 (10 µM) and 0.5 µL of Taq DNA polymerase (Invitrogen, Carlsbad, CA, USA. Optimized cycling conditions for the initial and the nested PCR reactions were as follows: initial PCR, 94 °C for 3 min followed by 40 cycles, repeating denaturation at 94 °C for 60 s, annealing at 58 °C for 60 s and elongation at 72 °C for 90 s, and a final extension step for 10 min at 72 °C; nested PCR 94 °C for 3 min followed by 5 cycles of 94 °C, 55 °C and 72 °C for 60 s each, and 35 cycles of 94 °C, 59 °C and 72 °C for 60 s each, and a final extension step of 10 min at 72 °C. Amplification products of the nested PCR were confirmed by electrophoresis in ethidium bromide-stained 2% agarose gel. PCR products were directly sequenced to identify sand fly-associated *Leishmania* species.

### 2.4. DNA Sequencing

The 462 bp nested PCR products obtained were purified using the ExoSAP-IT method using Exonuclease-I and Shrimp Alcaline Phosphatase, and sequenced in both directions using the Big Dye Terminator v3.1 kit (Applied Biosystems, Foster City, CA, USA with forward and reverse nested PCR primers (ITS1F/ITS2R4) [32]. Resulting consensus sequences were deduced by aligning the respective forward and reverse sequences using CLUSTAL_W 1.4 implemented in MEGA v.5.22 [33]. A total of 13 partial ITS-rDNA gene sequences, in addition to the studied sequences, were selected from Gene Bank database including two sequences from *L. infantum* (AJ000289, AJ000295), one sequence from *L. donovani* (AJ276260), three sequences from *L. tropica* (AJ300485, AJ000301, AJ000302), four sequences from *L. major* (AJ300482, AJ300481, AJ272383, AJ000310), two sequences from *L. turanica* (AJ272380, AJ272381), one sequence from *L. gerbilli* (AJ300486). Phylogenetic analysis was performed with MEGA v.5.22 software using the neighbor joining and the kimura-2 model. The tree topology was supported by 1000 bootstrap replicates.

## 3. Results

### 3.1. Sand Fly Species and Relative Abundance

A total of 4915 sand flies (2422 females and 2493 males) were collected from Saddaguia in September and October 2014. Morphological identification of sand files showed that *P. perfiliewi* was the most abundant species (50.8%), followed by *P. perniciosus* (23.5%), *P. longicuspis* (15.2%), and *P. papatasi* (9.7%). The remaining species were *Sergentomyia minuta* (*n* = 7) and *Sergentomyia dreyfussi* (*n* = 27). No atypical forms of *P. perniciosus* were observed.

### 3.2. Leishmania Infection in Sand Flies

A total of 170 pools of female sand flies were tested for *Leishmania* infection by nested PCR. Twenty-one pools representing female sand flies collected from houses and animal shelters within the peridomestic areas were found to be positive for *Leishmania* DNA. Of the sand fly pools positive for *Leishmania*, seven were of *P. perfiliewi*, five of *P. perniciosus*, four of *P. longicuspis*, four of *P. papatasi* and one of *S. minuta*. Hence, the overall minimum infection rate of sand flies with *Leishmania* DNA was 0.86% (21/2422). Sand flies giving positive result for *Leishmania* DNA were collected inside houses (bedrooms), and in chicken coops, rabbit holes, cattle pen, and sheep pen (Table 1).

### 3.3. Leishmania Sequencing and Phylogenetic Analysis

**Figure 3:**
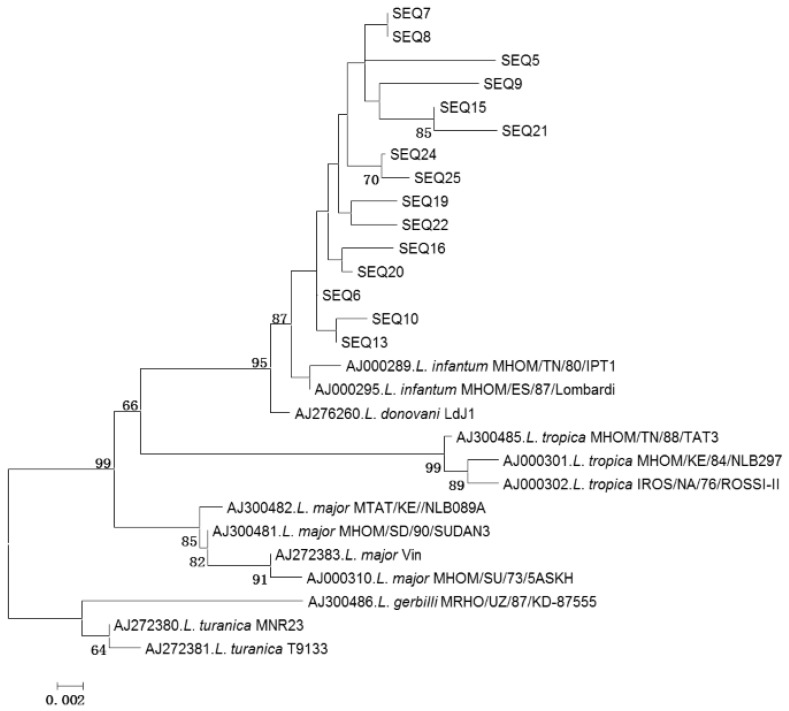
Phylogenetic tree based on partial *Leishmania* ITS-rDNA 5.8s sequences.

Of the 21 PCR products positive for *Leishmania* DNA, only 15 gave readable sequences. Identified *Leishmania* DNA sequences are closely related to the reference sequence of *L. infantum* AJ000289 *L. infantum* MHOM/TN/80/IPT1 isolated in Tunisia in 1980. Phylogenetic analysis showed that all 15 *Leishmania* sequences clustered together with the reference sequence of *L. infantum*. Phylogenetic branch including *L. infantum* sequences was supported by high bootstrap value (86%) (Figure 3). The overall infection rate of sand flies with *L. infantum* was 0.6% (15/2422). Infected pools consisted of three *P. perfiliewi*, four *P. perniciosus*, four *P. longicuspis*, three *P. papatasi* and one *S. minuta*.

The tree includes 15 *L. infantum* sequences reported in this study and 13 previously published representative *Leishmania* sequences species. Phylogenetic analysis was performed using neighbor joining and the kimura-2 parameter model. The tree topology was supported by 1000 bootstrap replicates. Bootstrap values lower than 60 were collapsed.

## 4. Discussion

Among a total of 17 sand fly species described in Tunisia [34], five were identified in the study area. *Phlebotomus perfiliewi* was the most abundant species followed by *P. perniciosus*, *P. longicuspis*, *P. papatasi*, *S. dreyfussi* and *S. minuta*.

*Phlebotomus perfiliewi* was known to be limited to the northern part of the Tunisian Ridge likely because of the high humidity and, likewise, is absent on the southern side of the Ridge due to the arid conditions [11,26,35,36]. Similar results were reported from Algeria [37,38]. In a transect study performed in 1980, a single *P. perfiliewi* specimen from the arid zone was reported [11]. Following a second transect performed in 2006, the overall abundance of *P. perfiliewi* in the governorate of Sidi Bouzid was 5% [18]. Our results suggest that *P. perfiliewi* is now the most abundant sand fly species in irrigated areas in Central Tunisia. It is important to note that *P. papatasi* is the most abundant sand fly species in the non-irrigated areas of the governorate of Sidi Bouzid [24]. Thus, the increase in the trapping rates of *P. perfiliewi* is consistent with the hypothesis of increased sand fly populations and risk of ZVL in arid areas following significant environmental changes due mainly to irrigation. Since *P. perfiliewi* has been found naturally infected with *L. infantum* in surrounding countries [39,40,41], it was considered as potential vector of *L. infantum* in Tunisia [35]. Here, we report for the first time the natural infection of *P. perfiliewi* with *L. infantum* in Tunisia based on the results obtained from the nested PCR analysis. Further, our analyses indicate an infection rate of *P. perfiliewi* with *L. infantum* of 0.12% (3/2422). Taken together, the high abundance of *P. perifiliewi* and the ability of this sand fly to be naturally infected with *L. infantum* provide yet further evidence for its role as vector of *L. infantum*.

*Phlebotomus perniciosus* is the principal vector of *L. infantum* in Tunisia, Algeria, and Italy [3,4,42]. *Phlebotomus perniciosus* was predominant north of the Tunisian Ridge and deemed rare in the south [26]. Thus, aridity appeared to be a limiting factor for its geographical distribution [26,38]. The entomological transects performed in 1980 and in 2006 indicated relative abundances of *P. perniciosus* in the arid bio-climatic zone to be 0.3% (*n* = 11,724) and 20% (*n* = 1024), respectively [11,18]. Thus, the geographical distribution of *P. perniciosus* is extending toward the center and the south with a concomitant increase in its relative abundance. Similar to the effects observed in *P. perfiliewi*, the increase in the trapping rates of *P. perniciosus* is consistent with the hypothesis of the pullulating sand flies and risk of ZVL following the development of irrigation in arid areas. *L. infantum-infected P. perniciosus* have been collected in the northern region and on the east coast of Tunisia [3,43]. Here, natural infection of *P. perniciosus* with *L. infantum* from the central part of the country is reported for the first time, and with an infection rate of 0.16% (4/2422).

*P. longicuspis* has also been found in all bio-geographical areas of Tunisia, from the humid north to the Sahara, with a predominance of 60% in the latter [18]. Similar results were reported from Southern Morocco where, *P. longicuspis* is the most abundant species in the arid bio-climatic zone and therefore, it is suspected to be the only vector of ZVL in this area [44,45]. *Phlebotomus longicuspis* was shown to be naturally infected with *L. infantum* in Algeria [46] and in Morocco [5]. This is the first report of natural infection of *P. longicuspis* with *L. infantum* in Tunisia and therefore, this sand fly species is considered a vector of *L. infantum*.

Sand flies of the genus *Sergentomyia* are vectors of reptile *Leishmania* [47]. As reported in Portugal [48], the presence of one pool of *S. minuta* infected with *L. infantum*, in our view, is not enough to conclusively incriminate this sand fly species as a vector. As reported in Greece and in Iran [49,50], the presence of *L. infantum* DNA in field-collected *P. papatasi* does not incriminate this sand fly species as a vector of ZVL. The presence of *L. infantum* DNA in *S. minuta* and *P. papatasi* could be explained by recent feedings on infected animals resulting in parasite DNA remnants after blood digestion.

Considering the five criteria usually required for a sand fly to be incriminated as a vector of leishmaniasis [51], our results provide good evidence that *P. perfiliewi* and *P. longicuspis* are indeed vectors of *L. infantum* in Saddaguia in addition to the already proven *P. perniciosus* [3]. In the present study, three main sand fly species of the subgenus *Larroussius* are involved in the transmission of *L. infantum.* The overall infection rate of sand flies with *L. infantum* is 0.6%. Similar infection rates were reported from the north and the east coast of Tunisia [3,43], and from surrounding countries [5,46,48]. The presence of infected sand fly species of the subgenus *Larroussius* inside houses (bedrooms) and animals shelter strongly suggests that these species are zoo-anthropophilic feeders and, subsequently, humans are at high risk for ZVL [5,40,43,48,52,53].

## 5. Conclusions

Based on our entomological findings, we conclude that the irrigation systems adopted in arid bio-geographical areas located in Central Tunisia have caused environmental changes that sustain populations of sand flies of the subgenus *Larroussius* and that this has led to the establishment of a stable transmission cycle of *L. infantum* and subsequently to the emergence of the ZVL focus. The continued increase in irrigated areas deserves attention, as it is associated with the spread of sand fly vectors of *L. infantum*. Thus, new vector control methods, such as attractive toxic sugar baits, show promise for reducing sand fly vector populations [54] and should be evaluated in light of our findings.

## Figures and Tables

**Table 1:** Detection of *Leishmania infantum* DNA in sand flies according to biotopes.

Code of Positive Pool	Date of Collection	Biotope	Number of Female Sandflies/Pool	*Leishmania* Species
Farm A				
T 3	4 September 2014	Bedroom	1. *P. perniciosus*	*L. infantum*
T 20	18 September 2014	Bedroom	3. *P. longicuspis*	*L. infantum*
T 23	18 September 2014	Bedroom	1. *S. minuta*	*L. infantum*
T 33	7 October 2014	Sheep shelter	1. *P. longicuspis*	*L. infantum*
T 36	7 October 2014	Chicken coop	2. *P. longicuspis*	*L. infantum*
Farm B				
T 61	18 September 2014	Sheep shelter	9. *P. perfiliewi*	*L. infantum*
T 63	7 October 2014	Bedroom	24. *P. perfiliewi*	*-*
T 68	7 October 2014	Sheep shelter	13. *P. perfiliewi*	*-*
T 72	21 October 2014	Sheep shelter	30. *P. papatasi*	*L. infantum*
T 74	21 October 2014	Sheep shelter	30. *P. perfiliewi*	*-*
Farm C				
T 8	4 September 2014	Rabbit hole	4. *P. papatasi*	*L. infantum*
T 93	4 September 2014	Chicken coop	30. *P. perfiliewi*	*L. infantum*
T 110	4 September 2014	Sheep and cattle shelter	30. *P. perfiliewi*	*-*
T 112	4 September 2014	Sheep and cattle shelter	1. *P. perniciosus*	*-*
T 128	18 September 2014	Sheep and cattle shelter	26. *P. perniciosus*	*L. infantum*
T 130	18 September 2014	Sheep and cattle shelter	9. *P. longicuspis*	*L. infantum*
T 131	18 September 2014	Sheep and cattle shelter	4. *P. papatasi*	*L. infantum*
T 133	18 September 2014	Bedroom	7. *P. perniciosus*	*L. infantum*
T 135	18 September 2014	bedroom	1. *P. papatasi*	*-*
T 140	7 October 2014	Rabbit hole	7. *P. perniciosus*	*L. infantum*
T 145	7 January 2014	Sheep shelter	12. *P. perfiliewi*	*L. infantum*

*-*: Sand flies were positive but the sequences were unredable.
